# γδ T Cell‐mediated Tumor Immunity is Tightly Regulated by STING and TGF‐β Signaling Pathways

**DOI:** 10.1002/advs.202404432

**Published:** 2024-11-21

**Authors:** Jing Luo, Shengli Wang, Quanli Yang, Qianqian Fu, Chuyun Zhu, Tao Li, Shuxian Yang, Yin Zhao, Rong Guo, Xiaosong Ben, Yuzhen Zheng, Sitao Li, Guang Yang, Hongru Zhang, Hui Xiao, Zhengfan Jiang, Nan Yan, Dieter Kabelitz, Guodong Sun, Zvi Granot, Ligong Lu, Fuping You, Jianlei Hao, Zhinan Yin

**Affiliations:** ^1^ Guangdong Provincial Key Laboratory of Tumor Interventional Diagnosis and Treatment Zhuhai Institute of Translational Medicine Zhuhai People's Hospital Affiliated with Jinan University Jinan University Zhuhai 519000 P. R. China; ^2^ State Key Laboratory of Bioactive Molecules and Druggability Assessment The Biomedical Translational Research Institute Health Science Center (School of Medicine) Jinan University Guangzhou 510632 P. R. China; ^3^ Key Laboratory of Viral Pathogenesis & Infection Prevention and Control (Jinan University) Ministry of Education Guangzhou 510632 P. R. China; ^4^ Institute of Reproductive Health Center for Reproductive Medicine Tongji Medical College Huazhong University of Science and Technology Wuhan 430030 P. R. China; ^5^ Department of Ophthalmology Tongji Hospital Tongji Medical College Huazhong University of Science and Technology Wuhan Hubei 430030 P. R. China; ^6^ Department of Obstetrics and Gynecology The First Affiliated Hospital of Gannan Medical University Ganzhou Jiangxi 341000 P. R. China; ^7^ Department of Thoracic Surgery Guangdong Provincial People's Hospital Guangdong Academy of Medical Sciences Guangzhou 510080 P. R. China; ^8^ Department of Pediatrics The Sixth Affiliated Hospital Sun Yat‐sen University Guangzhou 510655 P. R. China; ^9^ Department of Pathogen biology School of Medicine Jinan University Guangzhou 510632 P. R. China; ^10^ Nankai International Advanced Research Institute College of Life Sciences Nankai University Tianjin 300071 P. R. China; ^11^ Shanghai Institute of Immunity and Infection, Chinese Academy of Sciences Shanghai 200031 P. R. China; ^12^ Key Laboratory of Cell Proliferation and Differentiation of the Ministry of Education School of Life Sciences Peking University Beijing 100871 China; ^13^ Department of Immunology University of Texas Southwestern Medical Center Dallas TX 75390 USA; ^14^ Institute of Immunology University of Kiel and University Hospital Schleswig‐Holstein Campus Kiel 24105 Kiel Germany; ^15^ Guangdong Provincial Key Laboratory of Spine and Spinal Cord Reconstruction The Fifth Affiliated Hospital of Jinan University Heyuan 517000 P. R. China; ^16^ Department of Developmental Biology and Cancer Research Institute for Medical Research Israel Canada Hebrew University Medical School Ein Kerem Jerusalem 9112102 Israel; ^17^ Department of Immunology Institute of Systems Biomedicine School of Basic Medical Sciences Peking University Health Science Center Beijing 100191 P. R. China

**Keywords:** Eomes, IFN‐γ, STING, tumor immunity, γδ T cells

## Abstract

The STING pathway plays a critical role in tumor immunosurveillance. However, the precise mechanisms by which STING regulates gamma delta (γδ) T cell function during tumor progression remain unclear. Herein, we find that tumor‐derived cyclic GMP‐AMP (cGAMP) activates a distinct STING pathway by inducing TBK1‐mediated phosphorylation of Eomes in γδ T cells during the early stage of tumor development is demonstrated. This activation leads to interferon‐gamma (IFN‐γ) production and consequent tumor surveillance. However, at advanced stages of tumor progression, the accumulation of immune‐suppressive cytokine transforming growth factor‐beta (TGF‐β) downregulates STING levels, compromising the function of γδ T cells. Notably, the synergism between TGF‐β inhibition and STING agonists effectively counteracts the immunosuppressive tumor microenvironment, thereby augmenting the antitumoral effects of γδ T cells. These findings present a novel mechanism involving STING‐mediated IFN‐γ production in γδ T cells and hold significant implications for the development of potent immunotherapeutic approaches against cancer.

## Graphical abstract

Luo et al find that STING signaling is an important positive regulator of IFN‐γ production in γδ T cells in tumor immunity. The STING downstream kinase TBK1 binds Eomes to initiate IFN‐γ production. In late‐stage tumors, the STING‐IFN‐γ signaling in γδ T cells is inhibited by environmental TGF‐β. The finding provides potential therapeutics by STING activation and TGF‐β inhibition.

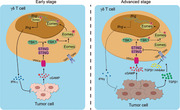

## 1 Introduction

Gamma delta (γδ) T lymphocytes represent a unique cell subset that is crucial in tumor immunity.^[^ ^1^ ^]^ We previously demonstrated that γδ T cells are essential as an early source of interferon‐gamma (IFN‐γ), a potent cytokine with anti‐tumor properties.^[^ ^2^ ^]^ Moreover, activated human Vδ2 γδ T cells have shown promising clinical efficacy against lung and liver cancers,^[^ ^3^ ^]^ highlighting their potential for cancer immunotherapy. However, although the importance of γδ T cells in tumor immunity is increasingly recognized, the underlying molecular mechanisms governing their anti‐tumor functions remain unclear.

The cytosolic DNA sensor, known as cGAS, generates cyclic dinucleotides that activate the stimulator of interferon genes (STING), triggering a signaling cascade that leads to the production of type I interferons (IFN‐Is) and pro‐inflammatory cytokines, initiating potent antiviral immune responses.^[^ ^4^, ^5^, ^6^ ^]^ Recent investigations have shed light on the involvement of STING in tumor immunity.^[^ ^7^, ^8^ ^]^ For example, STING signaling significantly impacts different types of immune cells, including CD4^+^ T,^[^ ^9^ ^]^ CD8^+^ T,^[^ ^10^ ^]^ natural killer (NK) cells, and B cells.^[^ ^11^ ^]^ The activation of STING–IFN‐I in antigen‐presenting cells (APCs) activates the tumor immune response.^[^ ^12^ ^]^ Moreover, IFN‐Is produced by APCs activate CD8^+^ T cells^[^ ^12^ ^]^ and NK cells^7^ in various mouse tumor models. Notably, STING agonists efficiently induce tumor rejection through IFN‐I‐activated CD8^+^ T cell responses^[^ ^13^ ^]^ or mechanisms involving NK cells.^[^ ^14^ ^]^ More recently, STING–IFN‐I signaling has been reported to promote the expression of T cell factor 1 (TCF1), a stem cell‐like T cell marker, and anti‐tumor function in CD8^+^ T cells.^[^ ^15^ ^]^ Additionally, intrinsic STING signaling in CD4^+^ T cells promotes Th1 and Th9 differentiation^[^ ^9^ ^]^ while inhibiting Th17 differentiation.^[^ ^16^ ^]^ In contrast, the loss of STING signaling in B cells increases tumor control.^[^ ^11^ ^]^ STING ligands also promote human Vδ2 γδ T cell activation and IFN‐γ production in vitro, which are APC‐dependent.^[^ ^17^ ^]^ However, the role of STING signaling in mouse γδ T cells and its implications for tumor immunity remain unclear.

Transforming growth factor‐beta (TGF‐β) exerts pleiotropic effects on various cell types within the tumor microenvironment.^[^ ^18^ ^]^ TGF‐β also modulates immune responses by suppressing the activity of effector immune cells and promoting the differentiation of regulatory T cells.^[^ ^19^, ^20^ ^]^ Moreover, TGF‐β promotes tumor immune evasion through various mechanisms, including the induction of immune checkpoint molecules.^[^ ^21^ ^]^ According to previous research, including work from our group, TGF‐β induces IL‐9 expression in human γδ  T cells^[^ ^22^ ^]^ and enhances cytotoxic effector function under serum‐free culture conditions.^[^ ^23^, ^24^ ^]^ However, the molecular mechanisms via which TGF‐β directly affects γδ T cells in the tumor microenvironment have not been fully elucidated.

Emerging evidence suggests a complex interplay between TGF‐β and STING signaling in the context of tumor progression.^[^ ^25^ ^]^ For example, TGF‐β can negatively regulate STING expression and activity, impairing the STING‐mediated anti‐tumor immune response.^[^ ^25^ ^]^ Conversely, STING activation can counteract the immunosuppressive effects of TGF‐β, thereby enhancing the efficacy of anti‐tumor immune responses.^[^ ^26^ ^]^ However, several clinical trials utilizing STING agonists have not been successful,^[^ ^27^, ^28^ ^]^ and the underlying mechanisms behind these failures remain unclear. Moreover, the precise activities of TGF‐β during cancer progression and the intricate interplay between TGF‐β and STING signaling in γδ T cells are not well defined.

Therefore, in this study, we aimed to elucidate the impact of dynamic changes in STING on γδ T cell function during cancer progression. To this end, we investigated the molecular mechanisms underlying γδ T cell‐mediated tumor immunity and explored the interplay among STING signaling, TGF‐β, and γδ T cells. Specifically, we sought to elucidate the mechanisms by which TGF‐β may hijack STING signaling in γδ T cells, thereby undermining their anti‐tumor function. Collectively, we believe that the findings of this study could shed light on a previously unrecognized mechanism employed by tumors to evade immune surveillance and promote tumor progression through interference with STING signaling by TGF‐β in γδ T cells.

## 2 Result

### 2.1 Tumor‐Derived cGAMP Activates γδ T Cells via STING–IFN‐γ Signaling in the Early Stage of Tumor Development

**Figure 1:**
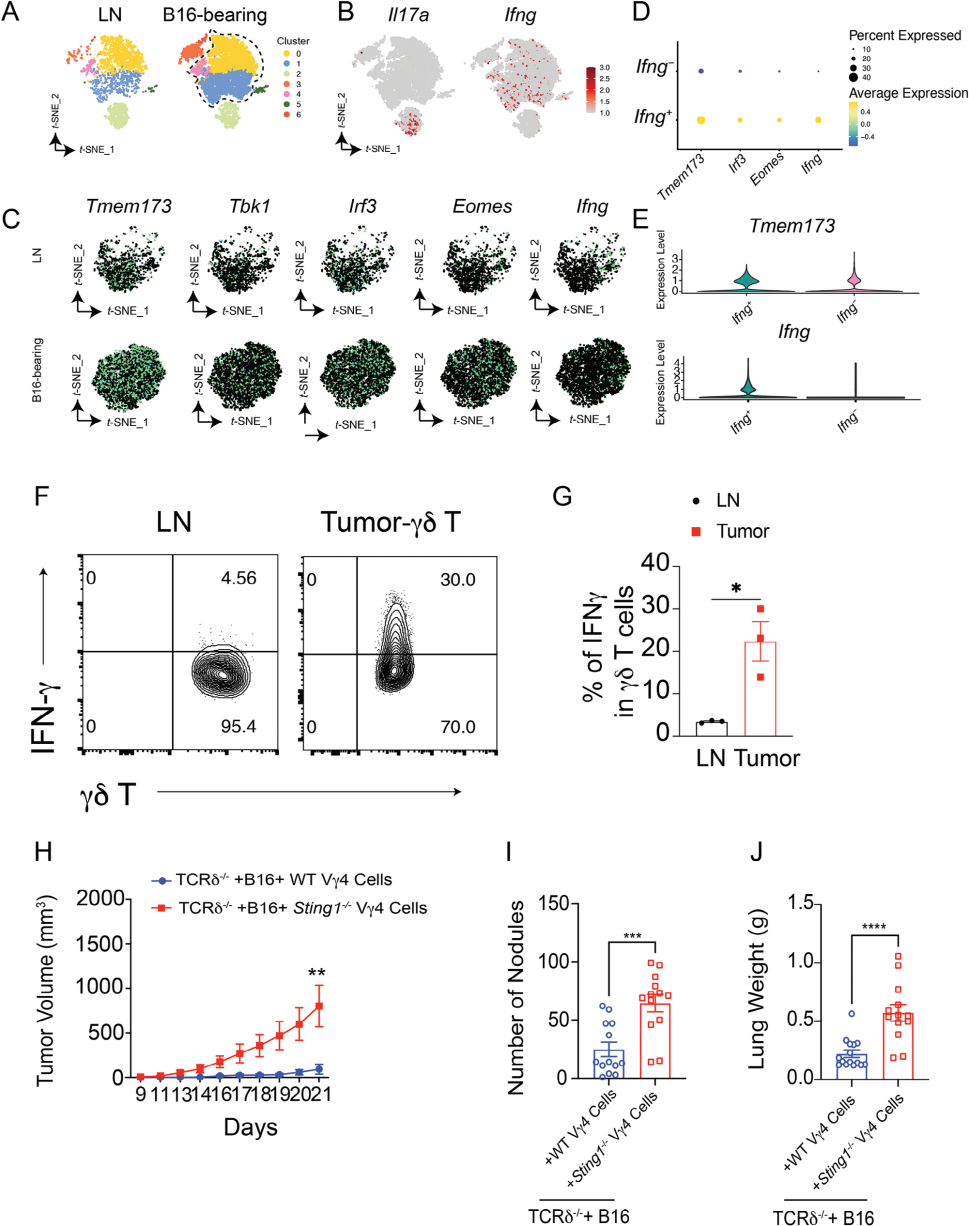
STING signaling promotes IFN‐γ production from tumor‐associated γδ T cells. A–C) Analysis of γδ T cells (CD45^+^CD3^+^γδ TCR^+^) isolated from tumor tissues of B16 tumor‐bearing mice on day 9 (*n* = 25) and lymph nodes (LNs) of naïve mice (*n *= 20) using scRNA‐seq. *T*‐distributed stochastic neighbor embedding (t‐SNE) plots show the expression of *Tmem173, Irf3, Tbk1, Eomes, and Ifng*. Each point represents a single cell colored according to cluster designation. B) γδ T cells are indicated, plotting *Ifng* ^+^ or *Il17a^+^ *γδ T cells. C) Expression of indicated genes on the *t‐*SNE plots from the data in (A) and (B). D) Functional enrichment analysis of the upregulated genes in *Ifng^+^* γδ T cells. E) Violin plots showing the expression levels of the indicated genes in *Ifng^+^* γδ T cells and *Ifng^−^* γδ T cells. F, G) IFN‐γ protein abundance in γδ T cells isolated from tumor tissues and LNs after B16 inoculation on day 9. Representative image (F) and quantification of IFN‐γ (G) in γδ T cells. H) Vγ4 γδ T cells from WT or *Sting1^−/−^* mice were expanded for 6 days, mixed with B16F0 cells, co‐injected into the flank of *TCRδ^−/−^* mice, and monitored for tumor growth. B16F0 tumor growth curves in *TCRδ^−/−^* mice co‐injected with Vγ4 γδ T cells of the indicated genotype are shown. I, J) Strategy for assessing the circulating Vγ4 γδ T cells in lung tumor surveillance. Vγ4 γδ T cells from WT (*n* = 13) or *Sting1* ^−/−^(*n* = 13) mice were expanded for 5–6 days and then adoptively transferred into the B16F0 bearing *TCRδ^−/−^* mice; mice were sacrificed for analysis post‐transfer day 21. The lungs of TCRδ^−/−^ mice for each group were isolated on day 21 post‐tumor injection; the numbers of tumor nodules (I) and lung weights (J) were measured. Each symbol represents an individual mouse. (K, L) Vγ4 γδ T cells (1 × 10^5^ cells) from WT or *cGAS^−/−^* mice were expanded for 5–6 days, mixed with B16F0 cells (4 × 10^5^ cells), and co‐injected into the flank of *TCRδ^−/−^* mice; tumor growth was monitored (*n* = 9 per group) (K). WT mice or *TCRδ^−/−^* mice were inoculated with B16 and *cGAS^−/−^* B16 cells and monitored for tumor growth (*n* = 9 per group) (L). Data are mean ± SEM. ***p ≤ 0.001; ns, not significant, two‐tailed unpaired *t‐*test. Data are representative of 3 independent experiments.

γδ T cells are critical in tumor immunosurveillance.^[^ ^2^, ^29^ ^]^ To investigate whether the anti‐tumor immunity mediated by γδ T cells is intrinsic or elicited by local tumor microenvironments, we compared γδ T cells residing in the lymph nodes with those infiltrating the tumor microenvironment during the early stage of tumor development in orthotopic B16 tumor‐bearing mice, a model characterized by potent γδ T cell infiltration.^2^ Functionally, γδ T cells (CD45^+^CD3^+^γδTCR^+^) isolated from early tumor tissues exhibited unexpectedly upregulated expression of *Tmem173, Irf3, Tbk1, Eomes*, and *Ifng* compared with that in their counterparts (**Figure** **1**A–C), in the lymph nodes, suggesting activation of the STING pathway in γδ T cells during the early stage of tumor progression. According to single‐cell RNA sequencing (scRNA‐seq) results, IFN‐γ^+^ γδ T cells showed higher expression levels of STING‐associated genes, including *Tmem173* and *Ifng*, than those in IFN‐γ^−^ γδ T cells isolated from B16 tumor‐bearing mice (Figure 1D,E). At the protein level, we observed notably higher expression of IFN‐γ in early tumor‐associated γδ T cells than in γδ T cells isolated from lymph nodes in B16 tumor‐bearing mice (Figure 1F,G).

As IFN‐γ is a potent anti‐tumor cytokine, we investigated whether STING activation enhanced γδ T cell‐mediated anti‐tumor immunity.^[^ ^30^ ^]^ We have previously demonstrated the critical role of the Vγ4 subsets of the mouse γδ T cells in γδ T cell‐mediated tumor killing through IFN‐γ.^[^ ^31^ ^]^ Therefore, we first compared the tumor‐killing capacity of STING‐activated Vγ4 γδ T cells with that of vehicle‐treated cells in vitro and found a markedly higher killing ability in the former than in the latter (Figure S1A,B, Supporting Information). To further confirm the critical role of IFN‐γ in Vγ4 γδ T cell anti‐tumor function, we added the STING agonist 5,6‐dimethylxanthenone‐4‐acetic acid (DMXAA) and an IFN‐γ‐neutralizing antibody to the co‐culture. IFN‐γ neutralization abolished the killing ability of Vγ4 γδ T cells (Figure S1A,B, Supporting Information). These findings suggest that exogenous STING activation potentiates Vγ4 γδ T cell anti‐tumor immunity through IFN‐γ.

To further confirm the potential role of endogenous STING signaling in regulating γδ T cell‐mediated tumor immunity, we applied the in vitro co‐culture system with wild‐type (WT) and *Sting1^−/−^* Vγ4 γδ T cells against a series of tumor cell lines. Notably, *Sting1^−/−^* Vγ4 γδ T cells exhibited significantly reduced killing capacity against Lewis lung carcinoma (LLC) (Figure S1C, Supporting Information), MC38 (Figure S1D, Supporting Information), and B16 (Figure S1E, Supporting Information) cells, suggesting that endogenous STING is required for γδ T cell‐mediated tumor inhibition in several cancer types.

To assess whether STING signaling in γδ T cells promoted the in vivo killing ability, we first used a subcutaneous co‐injection system, as described previously (Figure 1H).^[^ ^31^ ^]^ Vγ4 γδ T cells (WT or *Sting1^−/−^*) were mixed with B16 cells and injected into the flank of TCRδ^−/−^ mice. Results showed that the tumor nodules emerged earlier and tumors were larger in the *Sting1^−/−^* group (Figure 1H), indicating that endogenous STING activation promotes Vγ4 γδ T cell‐mediated anti‐tumor immunity in vivo.

To further investigate whether STING impacts the tumor surveillance of circulating Vγ4 γδ T cells in the lung, we transferred WT and *Sting1^−/−^* Vγ4 γδ T cells intravenously (*i.v*.) on day 1 post‐tumor injection and analyzed the lung tumor burden on day 21. *Sting1^−/−^* Vγ4 γδ T cells lost significant tumor‐killing function, as evidenced by increased metastasis (Figure 1I) and increased lung weight (Figure 1J). Moreover, RNA‐seq analysis revealed that STING activation induced a comprehensive type I response and ISGs in mouse γδ T cells but not in CD4^+^ or CD8^+^ T cells (Figure S2, Supporting Information). Comparably, RNA‐seq analysis revealed that CD8^+^ T cells exhibited upregulated TNF‐α expression upon DMXAA stimulation, which may have contributed to tumor immunity (Figure S3, Supporting Information). As NKG2D is a vital effector molecule in γδ T cells for antigen recognition and anti‐tumor immunity,^[^ ^32^ ^]^ we subsequently analyzed the effects of DMXAA and cGAMP on NKG2D expression in γδ T cells. However, no significant change was observed in NKG2D expression in γδ T cells upon STING signaling activation (Figure S4, Supporting Information). Collectively, the data suggest that STING activation potentiates Vγ4 γδ T cell anti‐tumor immunity by promoting IFN‐γ production.

STING is activated by cyclic dinucleotides such as cGAMP, which are synthesized upon cytosolic recognition of double‐stranded DNA by cGAS.^[^ ^33^ ^]^ Therefore, we analyzed the function of cGAS in γδ T cells. Unexpectedly, γδ T cell‐intrinsic cGAS was dispensable for anti‐tumor function (Figure S5A, Supporting Information), suggesting that γδ T cells incorporated environmental cGAMP during this process. Furthermore, we assessed the role of tumor‐derived cGAMP in eliciting immune responses in vivo. Notably, in *cGAS^−/−^* tumors, γδ T cells were dispensable for immune surveillance (Figure S5B, Supporting Information). Collectively, these data indicate that the γδ T‐mediated rejection of B16 tumors requires cGAMP from tumor cells and STING expression in γδ T cells.

### 2.2 Activation of STING Signaling Promotes IFN‐γ Secretion in γδ T Cells in a TBK1‐Activated Eomes‐Dependent Manner

**Figure 2:**
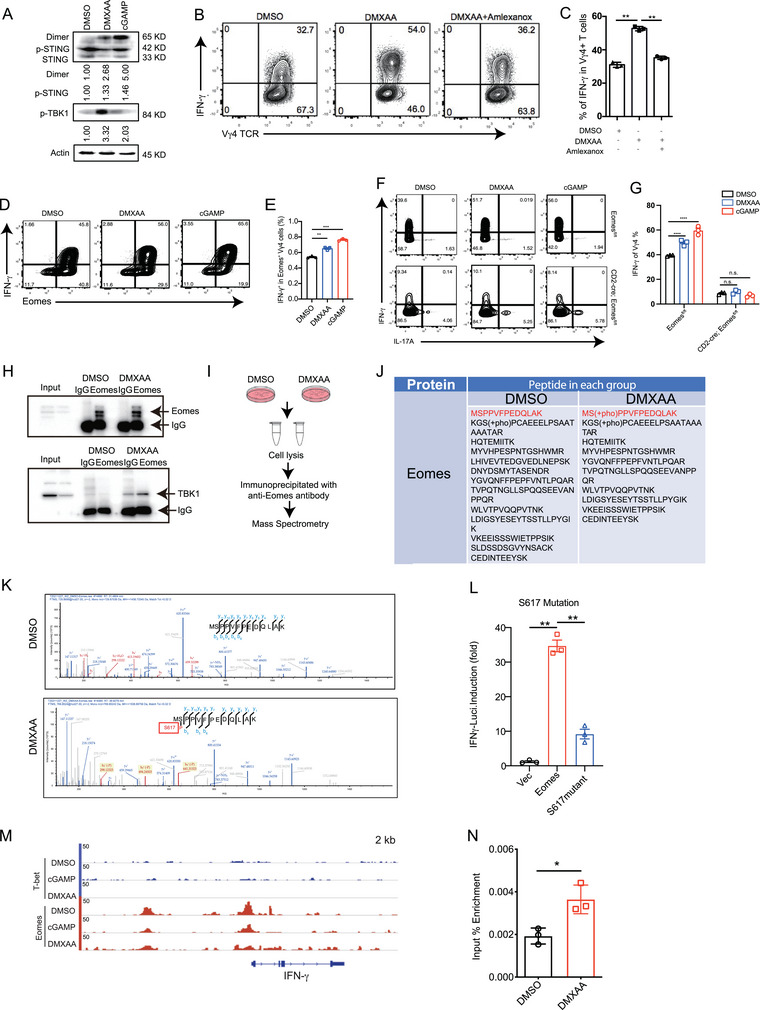
STING activates the TBK1–Eomes–IFN‐γ cascade in γδ T cells. A) Immunoblot analysis of p‐STING and p‐TBK1 expression in mouse Vγ4 γδ T cells after treatment with dimethyl sulfoxide (DMSO) or DMXAA (10 µg mL^−1^) for 6 h or cGAMP (5 µg mL^−1^) for 24 h. B, C) WT Vγ4 γδ T cells were expanded for 6 days (purity > 90%) and treated with DMXAA with or without the TBK1 inhibitor amlexanox (15 µM) for 6 h, cells were re‐stimulated with PMA and ionomycin in the presence of GolgiPlug for 4 h, and intracellular IFN‐γ was stained and analyzed. Representative staining (B) and quantification (C) of IFN‐γ in Vγ4 γδ T cells. D, E) WT Vγ4 γδ T cells were expanded for 6 days (purity > 90%) and treated with DMXAA (10 µg mL^−1^) for 6 h and cGAMP (5 µg mL^−1^) for 24 h; DMXAA‐ or cGAMP‐treated cells were re‐stimulated with PMA and ionomycin in the presence of GolgiPlug for 4 h and intracellular IFN‐γ and Eomes were stained. (D) Representative staining and (E) relative abundance of IFN‐γ in Eomes^+^ Vγ4 γδ T cells. F, G) Vγ4 γδ T cells from *Eomes^f/f^* or *CD2^cre^‐Eomes^f/f^* mice were expanded for 6 days (purity > 90%) and treated with DMXAA (10 µg mL^−1^) for 6 h and cGAMP (5 µg mL^−1^) for 24 h; cells were re‐stimulated with PMA and ionomycin in the presence of GolgiPlug for 4 h, and intracellular IFN‐γ was stained and analyzed. Representative staining (F) and quantification (G) of IFN‐γ in Vγ4 γδ T cells. H) WT Vγ4 γδ T cells were expanded for 6 days (purity > 90%) and treated with DMXAA (10 µg mL^−1^) for 6 h; cell lysates were incubated with anti‐Eomes; an Eomes IP (upper panel) and TBK1 IP (lower panel) are shown. I) Eomes protein phosphorylation analysis via mass spectrometry. WT Vγ4 γδ T cells were expanded for 6 days (purity > 90%) and treated with DMXAA for 6 h; cell lysates were incubated with an anti‐Eomes antibody for immunoprecipitation and mass spectrometry. J, K) Eomes phosphorylation site after DMXAA treatment. L) Luciferase reporter assay of Eomes‐induced transcription of IFN‐γ after phosphorylation site (Ser617) mutation. M, N) T‐bet and Eomes ChIP‐seq performed on WT Vγ4 γδ T cells expanded for 6 days (purity > 90%) and treated with DMXAA (10 µg mL^−1^) for 6 h or cGAMP (5 µg mL^−1^) for 24 h. T‐bet and Eomes binding peaks at *Ifng* (M) and enrichment relative to input (N). Data are mean ± SEM. *p ≤ 0.05, **p ≤ 0.01, ***p ≤ 0.001; ns, not significant, two‐tailed unpaired *t‐*test. Data are representative of 3 independent experiments.

To confirm that cytokine induction by DMXAA treatment is specific to STING, we expanded γδ T cells from WT and *Sting1^−/−^* mice and treated them with DMXAA. We observed that the response of *Sting1^−/−^* mice to DMXAA and cGAMP treatment was reversed (Figure S6, Supporting Information). These data suggest that DMXAA‐ and cGAMP‐induced IFN‐γ expression in γδ T cells is fully dependent on STING. Furthermore, γδ T cells from STING S365A mutant mice exhibited no IFN‐γ response following cGAMP treatment only and not following treatment with DMXAA (Figure S7, Supporting Information). Similar to dendritic cells and macrophages, mouse γδ T cells demonstrated activation of p‐STING and p‐TBK1 upon STING activation (**Figure** **2**A). cGAS^−/−^ γδ T cells exhibited a similar response (upregulation of IFN‐γ) to DMXAA treatment when compared with WT cells (Figure S8A, Supporting Information). Moreover, DMXAA did not increase IFN‐γ levels in Vγ4 γδ T cells pretreated with the TBK1 inhibitor amlexanox, suggesting that TBK1 is required for STING‐induced IFN‐γ production (Figure 2B,C). Consistently, the mRNA levels of *Ifna*, *Ifnb*, and *Ifng* were all reduced following amlexanox treatment in the presence of DMXAA (Figure S8B–D, Supporting Information).

Consistent with previous findings,^[^ ^31^, ^34^ ^]^ we also observed that IFN‐γ was significantly induced in Eomes‐positive cells (Figure 2D,E), indicating that Eomes was activated by the STING agonist. Furthermore, IFN‐γ induction by DMXAA and cGAMP was completely abolished in Eomes‐conditional KO γδ T cells (Figure 2F,G). However, Vγ4 γδ T cells from T‐box factor T‐bet‐knockout mice retained responsiveness to DMXAA, as evidenced by increased IFN‐γ production after treatment with DMXAA and cGAMP (Figure S9, Supporting Information). To further identify the potential direct interaction between TBK1 and Eomes, a co‐immunoprecipitation assay was performed, revealing direct binding between endogenous Eomes and TBK1 in γδ T cells upon treatment with DMXAA (Figure 2H). Additionally, immunoprecipitation of endogenous Eomes was performed using an anti‐Eomes antibody in Vγ4 γδ Τ cells, followed by mass spectrometric analysis. Notably, the Eomes Ser617 site was phosphorylated in Vγ4 γδ Τ cells after treatment with DMXAA (Figure 2I–K). Hence, the significance of the Ser617 site was evaluated by substituting the serine with alanine (S617A). The luciferase reporter assay revealed that mutation of this site abrogated the induction of IFN‐γ by DMXAA (Figure 2L), suggesting that phosphorylation of Eomes at Ser617 is required for transcriptional activation of IFN‐γ. To examine if Eomes was bound to the promoter region of IFN‐γ in response to DMXAA, we immunoprecipitated the cell lysate of Vγ4 γδ Τ cells with the Eomes antibody and performed genome‐wide chromatin immunoprecipitation and sequencing (ChIP‐seq) analysis, with T‐bet as a control. Consistently, Eomes, rather than T‐bet, bound to the *Ifng* promoter in Vγ4 γδ Τ cells after treatment with DMXAA or cGAMP (Figure 2M). Indeed, DMXAA enhanced the binding of Eomes to the *Ifng* promoter (Figure 2N). Collectively, these results suggest that the activation of STING signaling in γδ T cells promotes IFN‐γ secretion and is dependent on TBK1‐activated Eomes.

### 2.3 The STING–IFN‐γ Axis Mediates Antitumor Immunity and Inhibits Exhaustion in Human Vδ2 γδ T Cells

**Figure 3:**
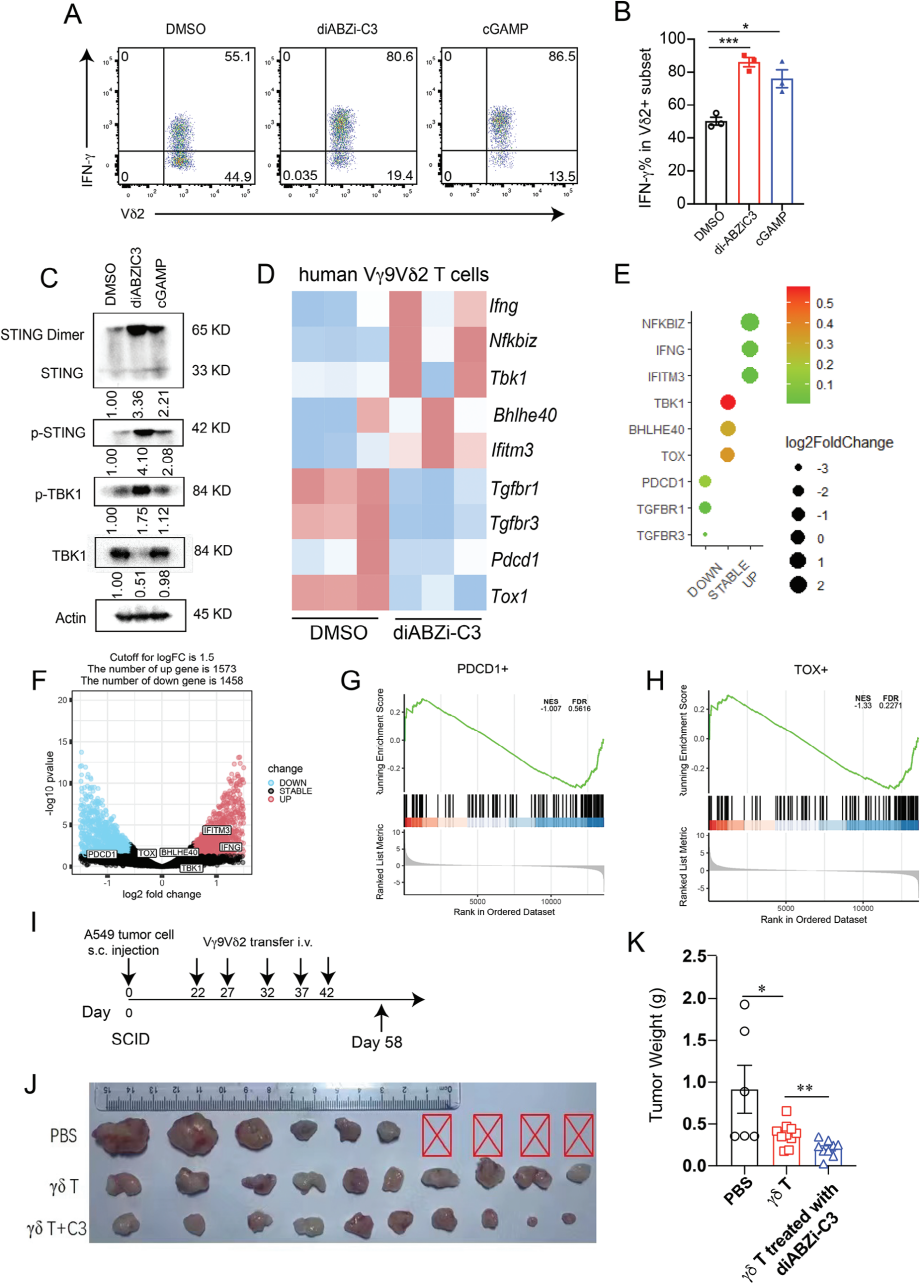
STING is important for human Vδ2 γδ T cell IFN‐γ production and anti‐tumor function. A, B) Human γδ T cells were expanded for 12 days (purity > 90%) and were treated with DMSO or diABZI‐C3(100 nM) for 6 h and cGAMP (5 µg mL^−1^) for 24 h. Cells were re‐stimulated with PMA and ionomycin in the presence of GolgiPlug for 4 h, and intracellular IFN‐γ was stained. Representative staining (A) and quantification of IFN‐γ in human γδ T cells (B). C) Immunoblot analysis of p‐STING and p‐TBK1 expression in human γδ T cells after DMSO, diABZI‐C3, or cGAMP treatment for 6 h, respectively. D) Heatmaps showing the relative transcript level of representative genes in DMSO‐ and diABZiC3‐treated human γδ T cells. Asterisks represent transcripts with significant differential expression (FDR < 0.05 and log2FoldChange > 1). E) GO enrichment analysis showing representative biological pathways for up‐ and downregulated genes in DMSO‐ and diABZiC3‐treated human γδ T cells. q values (columns) are presented as −log10(q); circles: gene ratios (%). ATP, adenosine 5′‐triphosphate. F) Volcano plots showing differentially expressed genes in human γδ T cells (Fold change > 1.5) between the diABZi‐C3 group and control group. G, H) GSEA for comparing the enrichment of PDCD^+^ and TOX^+^ human γδ T cell signature genes in the transcriptomes of DMSO‐ and diABZiC3‐treated human γδ T cells. I) Establishing A549 tumors in SCID mice and evaluating the therapeutic effects of diABZi‐C3 (100 nM) on Vγ9Vδ2 γδ T cells. At 22 days after inoculation of A549 cells (5 × 10^6^ cells per mouse) into SCID mice, Vγ9Vδ2 γδ T cells treated with or without diABZi‐C3 were adoptively transferred i.v. Into SCID mice. Mice treated with PBS were used as the control (*n* = 10 per group). Tumors for each group were isolated on day 55 post‐tumor injection J); tumor weight K). Data are mean ± SEM. **p ≤ 0.01, ***p ≤ 0.001, ****p ≤ 0.0001; ns, not significant, two‐tailed unpaired *t‐*test. Data are representative of 3 independent experiments.

To further determine the role of STING–IFN‐γ signaling in human Vγ9Vδ2 γδ T cells, we treated human Vγ9Vδ2 γδ T cells from peripheral blood mononuclear cells (PBMCs) with the STING agonist diABZi‐C3 and analyzed cytokine production. Consistently, human Vγ9Vδ2 γδ T cells produced IFN‐γ upon STING activation with diABZi‐C3 or cGAMP treatment (**Figure** **3**A,B). We then examined the downstream signaling of STING in human γδ T cells and observed activation of p‐STING and p‐TBK1 upon STING activation (Figure 3C). Additionally, RNA‐seq analysis revealed that *IFNG*, *TBK1*, and *NFKB* were upregulated, whereas the exhaustion markers *PDCD1* and *TOX1* and the TGF‐β receptor family *TGFR1* and *TGFR3* were significantly inhibited upon STING activation in human Vδ2 γδ T cells (Figure 3D–H). To investigate whether STING activation promoted human γδ T cell anti‐tumor function, we utilized an applied adoptive transfer model of severe combined immunodeficiency disease (SCID) mice. First, A549 lung cancer cells were injected into SCID mice, and human Vγ9Vδ2 γδ T cells pretreated with or without diABZi‐C3 were sequentially transferred 5 times (Figure 3I). Mice receiving diABZi‐C3‐pretreated Vγ9Vδ2 γδ T cells exhibited significantly enhanced anti‐tumor activity (Figure 3J,K). Collectively, human Vγ9Vδ2 γδ T cells displayed significant inhibition of tumor progression upon STING activation. These results highlight the importance of the STING–IFN‐γ axis in mediating the anti‐tumor function of human Vδ2 γδ T cells.

### 2.4 TGF‐β and the STING Pathway are Inversely Correlated During Tumor Progression

**Figure 4:**
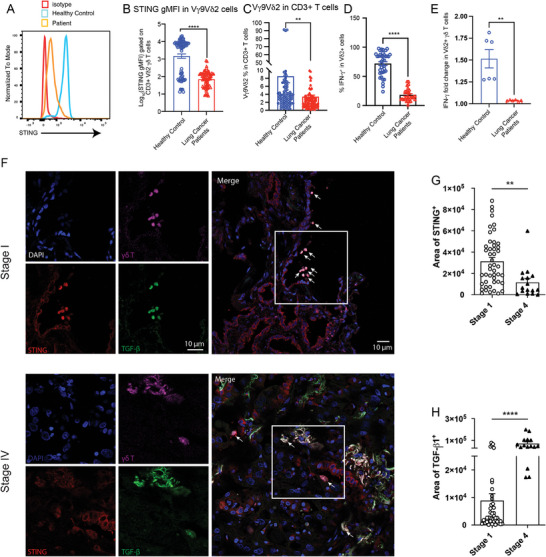
TGF‐β1 expression is inversely correlated with STING in γδ T cells and with survival in patients with lung adenocarcinoma. A, B) Representative histogram (A) and mean fluorescence intensity (MFI) summary (B) showing STING expression in Vδ2 γδ T cells from the peripheral blood of healthy volunteers (*n* =70) and patients with lung cancer (*n* = 75). C) Quantification of the percentage of Vδ2 γδ T cells in the peripheral blood of healthy volunteers (*n* =97) and patients (*n* =90). D) Quantification of IFN‐γ^+^ in Vδ2 γδ T cells from the peripheral blood of healthy volunteers (*n* =38) and patients with lung cancer (*n* =27). E) Fold change in intracellular IFN‐γ levels in cultured human Vδ2 γδ T cells from patients with lung cancer (*n* =6) and control healthy individuals (*n* =6) treated with diABZi‐C3 (100 nM) for 6 h. F–H) A representative immunofluorescence staining image of 4′,6‐diamidino‐2‐phenylindole [DAPI (blue)], anti‐STING (red), and anti‐TGF‐β (green) in human primary lung adenocarcinoma tissues. A representative immunostaining image of DAPI (blue), anti‐STING (red), and anti‐TGF‐β (green) in human lung tumor tissues (F). Quantification of the STING^+^ area percentage (G) and TGF‐β1^+^ area percentage (H) in the tumor areas. Data are the mean ± SEM. *p ≤ 0.05, ***p ≤ 0.001 (two‐tailed unpaired *t‐*test). Data are representative of 3 independent experiments.

We previously demonstrated the activation of the STING–IFN‐γ pathway during the early stages of tumor development, prompting inquiry into how tumors eventually evade this response. We hypothesized that decreasing STING expression levels in γδ T cells are associated with cancer progression. To validate our hypothesis, we examined STING/IFN‐γ expression in cultured Vδ2 γδ T cells from patients with lung cancer and observed significantly lower STING expression in lung cancer patients than in healthy donors (**Figure** **4**A,B). Consistently, IFN‐γ levels were also diminished in the γδ T cells of the patients (Figure 4C,D), and IFN‐γ induction was severely impaired in response to diABZI‐C3 (Figure 4E). This confirmed the functional deficiency of STING in cancer. TGF‐β reportedly downregulates STING expression, and STING supports IFN‐β and CCL5 levels.^[^ ^25^ ^]^ TGF‐β exhibited limited effects on cGAS or cGAMP levels,^[^ ^25^ ^]^ suggesting that increased levels of TGF‐β in the late tumor stage might induce STING downregulation. Here, we observed that stage IV tumor tissue samples exhibited few γδ T cells and low levels of STING, whereas stage I tumor tissues contained a significant number of γδ T cells and high levels of STING (Figure 4F,G). Conversely, TGF‐β1 expression was upregulated in stage IV compared with that in stage I tumor tissues (Figure 4F,H). Collectively, these data suggest that inhibition of STING activity in γδ T cells via increased TGF‐β1 levels may be associated with survival in patients with human lung adenocarcinoma. In conclusion, the expression of STING–IFN‐γ in γδ T cells is inversely correlated with TGF‐β1 expression and malignant tumor grade in patients with lung adenocarcinoma.

### 2.5 STING–IFN‐γ Signaling in γδ T Cells is Impaired During Tumor Progression in a Mouse Model

**Figure 5:**
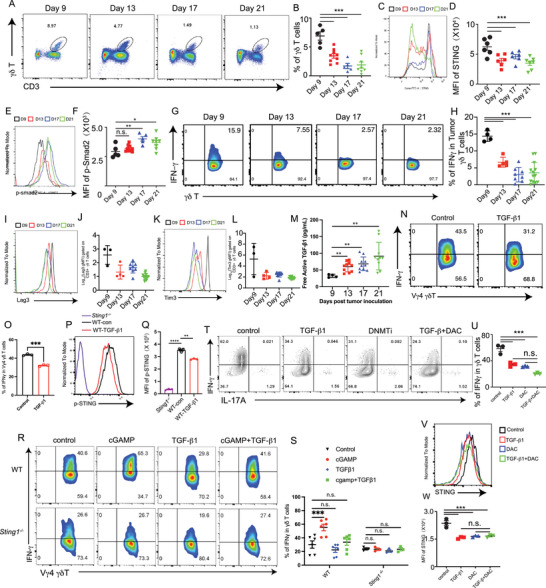
STING–IFN‐γ signaling in γδ T cells is impaired during tumor progression in mice. A–I) WT mice were inoculated with B16 cells (4 × 10^5^), and tumor growth was monitored on days 9, 13, 17, and 21. (A) Representative staining and (B) quantification of γδ T cells. Representative histogram (C) and mean fluorescence intensity (MFI) summary (D) showing p‐STING expression in γδ T cells from tumor‐infiltrating lymphocytes (TILs) in each group. Representative histogram (E) and MFI summary (F) showing p‐Smad2 expression in γδ T cells from TILs per group. Representative staining (G) and quantification (H) of IFN‐γ^+^ in γδ T cells. Representative histogram (I) and MFI summary J) showing Lag3 expression in γδ T cells from TILs. Representative histogram K) and MFI summary L) showing Tim‐3 expression in γδ T cells from TILs. M) ELISA was performed to determine the protein levels of TGF‐β1 in the tumor tissues in each group. N–Q) WT Vγ4 γδ T cells were expanded for 6 days (purity > 90%), treated with DMSO and TGF‐β1 (0.5 ng mL^−1^) for 24 h, and re‐stimulated with PMA and ionomycin in the presence of GolgiPlug for 4 h. Representative staining (N) and quantification (O) of IFN‐γ in Vγ4 γδ T cells. Representative histogram (P) and MFI summary (Q) showing p‐STING expression in γδ T cells. R, S) Vγ4 γδ T cells from *WT* or *Sting1^−/−^* mice were expanded for 6 days (purity > 90%), treated with TGF‐β1 and cGAMP (5 ng mL^−1^) for 24 h, and re‐stimulated with PMA and ionomycin in the presence of GolgiPlug for 4 h. Representative staining (R) and quantification (S) of IFN‐γ in Vγ4 γδ T cells. T–W) WT Vγ4 γδ T cells were expanded for 6 days (purity > 90%), treated with TGF‐β1 (0.5 ng mL^−1^) with or without DAC for 24 h, and re‐stimulated with PMA and ionomycin in the presence of GolgiPlug for 4 h. Representative staining (T) and quantification (U) of IFN‐γ in Vγ4 γδ T cells. Representative histogram (V) and mean fluorescence (W). Data are mean ± SEM. *p ≤ 0.05, ***p ≤ 0.001 (two‐tailed unpaired *t‐*test). Data are representative of 3 independent experiments.

To further evaluate the expression levels of STING and the functional states of γδ T cells in the mouse model, we conducted fluorescence‐activated cell sorting (FACS) assays on tumor‐infiltrating γδ T cells on days 9, 13, 17, and 21 post‐tumor‐cell inoculation. Similar to the findings in humans (Figure 4F–H), the number of tumor‐infiltrating γδ T cells decreased during tumor progression (**Figure** **5**A,B). Additionally, these tumor‐infiltrating γδ T cells exhibited reduced expression of STING (Figure 5C,D) and upregulated p‐Smad2 (Figure 5E,F) in tumor tissues as the tumor progressed. Furthermore, the expression of the effector cytokine IFN‐γ was reduced, and several exhaustion markers, including Lag‐3, and Tim‐3, in tumor‐infiltrating γδ T cells, indicating the presence of an exhaustion‐like phenotype due to prolonged tumor burden (Figure 5G–L). However, the expression of active TGF‐β1 in tumor tissues was upregulated with tumor progression (Figure 5M).

Subsequently, we investigated the mechanisms by which TGF‐β impacts the production of IFN‐γ and STING levels in γδ T cells. Treatment of γδ T cells with TGF‐β1 in culture resulted in significant downregulation of IFN‐γ and p‐STING levels (Figure 5N–Q). Moreover, *Sting1^−/−^* γδ T cells lacked the IFN‐γ response to TGF‐β (Figure 5R,S), confirming the involvement of STING signaling in the process. To further investigate the mechanisms underlying the TGF‐β‐mediated downregulation of STING–IFN‐γ signaling, we explored the mechanisms involving methylation of the Sting1 promoter. To this end, the methylation transferase inhibitor DAC (decitabine, DNMTi) was added to the TGF‐β‐treated γδ T cell culture. FACS results suggested that following DAC treatment, TGF‐β lost its ability to downregulate STING and inhibit cGAMP‐mediated IFN‐γ production (Figure 5T–W). Therefore, TGF‐β likely downregulates STING levels in γδ T cells through methylation‐dependent mechanisms.

### 2.6 TGFβR Blockade in γδ T Cells Improves STING Agonist Efficacy in the Immunotherapy of Late‐Stage Tumors

**Figure 6:**
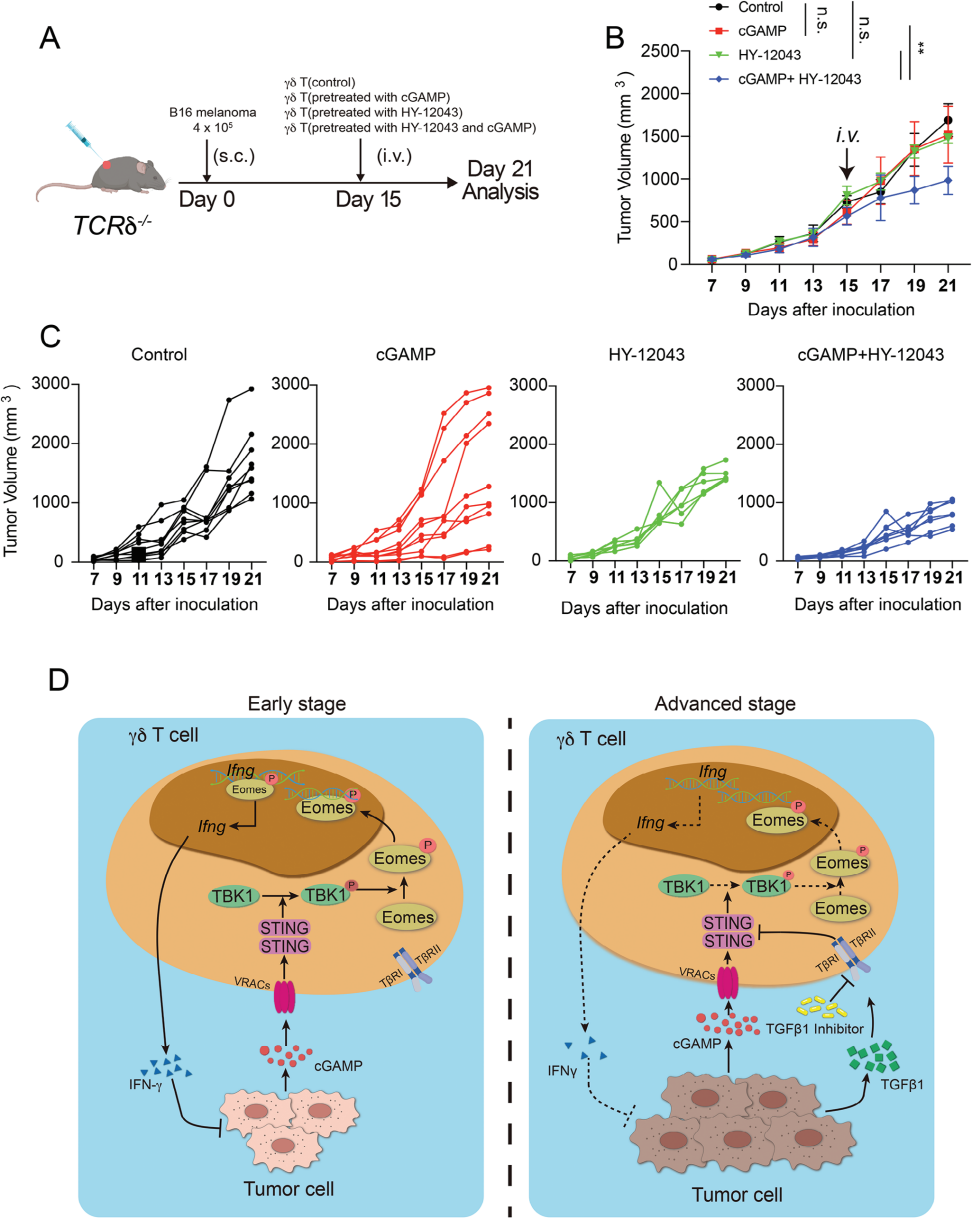
TGF‐β1 inhibits STING‐dependent anti‐tumor immunity of γδ T cells. (A–C) Establishment of B16 tumors in *TCRδ^−/−^* mice and evaluation of the effects of treating γδ T cells for 24 h with cGAMP (5 µg mL^−1^), TGFbR inhibitor (2 µM) alone, or their combination. At 15 days post‐inoculation of B16 cells (4 × 10^5^ cells/mouse) into *TCRδ^−/−^* mice, γδ T cells pretreated with cGAMP, HY‐12043, or cGAMP with TGFbR inhibitor was adoptively transferred i.v^.^ Into the tumor‐bearing *TCRδ^−/−^* mice. Mice treated with PBS were used as the control (*n* = 7–10/group). Average growth curve (B) and individual tumor growth curves (C). (D) Graphical abstract. Data are mean ± SEM. **p ≤ 0.01, ***p ≤ 0.001, ****p ≤ 0.0001; ns, not significant, two‐tailed unpaired *t‐*test. For tumor size comparison, two‐way ANOVA was applied. Data are representative of 3 independent experiments.

Considering the downregulated STING expression in γδ T cells and increased levels of TGF‐β in late‐stage tumors, we further explored the potential application of combining TGF‐β inhibition with STING activation in γδ T cell‐mediated immunotherapy for late‐stage tumors. To this end, we pretreated γδ T cells with the TGF‐β1R inhibitor in the presence of cGAMP and then transferred them (i.v.) into *TCR*δ*^−/−^* mice on day 15 post‐tumor inoculation and observed tumor rejection (**Figure** **6**A). Notably, cGAMP treatment alone did not alter tumor growth (Figure 6B,C), suggesting a dispensable role for STING in the anti‐tumor function of γδ T cells in late‐stage tumors, which differs from that in the early stage (Figure 1). Furthermore, a combination of TGF‐β1R signaling blockage and cGAMP treatment could control tumor growth significantly (Figure 6B,C), confirming that elevated TGF‐β levels hijacked γδ T cell effector function during the late stage of tumor progression.

## 3 Discussion

Several studies have confirmed that γδ T cells play a critical role in tumor immunity by providing an early source of IFN‐γ.^[^ ^2^, ^3^ ^]^ In fact, adoptive transfer of in vitro expanded human Vδ2 γδ T cells has shown promising clinical safety and efficacy.^[^ ^35^ ^]^ However, the detailed molecular mechanisms responsible for IFN‐γ production and negative regulation of γδ T cells leading to tumor evasion are not fully understood. In this study, we confirmed that tumor‐derived cGAMP activates γδ T cells through the STING–IFN‐γ signaling pathway during early tumor development. Notably, we observed an inverse correlation between STING–IFN‐γ expression and TGF‐β levels in γδ T cells during tumor progression. We also elucidated the role of TGF‐β as a key regulator that downregulates IFN‐γ and STING and induces exhaustion markers in γδ T cells. Finally, we demonstrated that blocking TGF‐β receptors restores STING function and enhances the efficacy of STING agonists in late‐stage tumor immunotherapy mediated by γδ T cells.

Hence, we have elucidated a previously unknown mechanism involving STING‐mediated TBK1 and Eomes phosphorylation, which is essential for the production of IFN‐γ in γδ T cells. CD4 T cells show no Eomes expression. Although CD8 T cells express Eomes, the activation state of Eomes–IFN‐γ signaling in these cells is lower than that in γδ T cells. STING activation reportedly induces IFN‐γ production in human Vδ2 γδ T cells in vitro; however, the responsiveness to STING ligands appears to differ between resting and activated γδ T cells17; however, the detailed mechanisms underlying the process remain undefined. By employing immunoprecipitation and knockout mouse models, we identified Eomes and the Ser617 phosphorylation site as requirements for activating IFN‐γ production (Figure 2). Additionally, we demonstrated the indispensable role of STING phosphorylation using STING S365 mutant transgenic mice (Figure S6, Supporting Information). The STING S365 residue is required to recruit the transcription factor IRF3 to TBK1.^[^ ^35^, ^36^ ^]^ Additionally, S365 is required for STING‐mediated IFN‐γ signaling through the TBK1–Eomes axis in γδ T cells. This suggests that STING may use the same signaling platform to recruit and activate divergent transcription factors in different cell types. Therefore, our findings provide crucial insights into the signaling events that lead to IFN‐γ production in γδ T cells upon STING activation. However, the involvement of IFN‐I signaling after STING activation requires further investigation to characterize the signaling cascades triggered by STING activation in γδ T cells.

Our findings also highlight the potent tumor immunity conferred by the STING–IFN‐γ signaling pathway during the early stages of tumor development. This observation is supported by studies using mice and human samples of peripheral Vδ2 γδ T cells. Activation of STING in γδ T cells increases the mRNA levels of *TBK1, Eomes, and IFN‐Υ*, exerting anti‐tumor effects in a STING‐dependent manner. Notably, cGAS‐deficient γδ T cells exhibited no difference in STING activation, whereas cGAS‐deficient tumor cells failed to be eradicated by γδ T cells (Figure 1). This suggests that cGAS‐dependent cGAMP from tumor cells activates STING in γδ T cells and mediates potent tumor immunity during the early stages of tumor development.

Our findings shed light on the interplay between TGF‐β and STING signaling during the late stages of tumor development. This interplay provides a potential explanation for the suppression of γδ T cell function observed during tumor progression. In particular, we demonstrated an inverse correlation between STING–IFN‐γ expression in γδ T cells and TGF‐β levels in tumor tissues, highlighting the involvement of TGF‐β in modulating the immune responses of γδ T cells. We have also provided experimental evidence for TGF‐β inhibiting STING expression levels in γδ T cells through a methylation‐dependent mechanism, leading to decreased IFN‐γ production and compromised STING‐mediated tumor inhibition (Figure 5). The findings demonstrate the importance of considering the inhibitory effects of TGF‐β on γδ T cells when designing immunotherapeutic strategies targeting the STING pathway. To the best of our knowledge, the mechanisms by which TGF‐β targets γδ T cell STING expression levels in a methylation‐dependent manner have not been reported previously and might account for γδ T cell exhaustion in the tumor microenvironment.

Finally, we demonstrated that by inhibiting TGF‐β using the TGF‐β1 receptor inhibitor, STING function can be restored and the efficacy of STING agonists could be enhanced (Figure 6). Therefore, blocking TGF‐β signaling in combination with STING antagonists has the potential to introduce effective immunotherapeutic interventions against late‐stage cancer. However, further studies are warranted to evaluate its potency and efficacy in a broader range of tumor models.

### 3.1 Limitations

Our study had certain limitations. First, we predominantly focused on murine models, and while human samples are included, further validation in human clinical settings is necessary to confirm the translational relevance of the findings. Second, we predominantly investigated the early and late stages of tumor development, leaving a gap in understanding the dynamics of STING–IFN‐γ signaling and TGF‐β regulation during intermediate stages. Third, the mechanisms underlying the crosstalk between STING signaling and other immune checkpoints or pathways, such as PD‐1/PD‐L1, remain largely unexplored. Fourth, the study primarily emphasizes the role of γδ T cells in tumor immunity, but it would be valuable to investigate potential interactions with other immune cell populations within the tumor microenvironment, such as regulatory T cells or myeloid‐derived suppressor cells. In addition, further direct biochemical evidence such as an in vitro kinase assay is needed to confirm that TBK1 interacted and phosphorylated Eomes. Finally, while the combination therapy approach of TGF‐β blockade and STING activation shows promising results in late‐stage tumors, the long‐term effects and potential adverse events of this treatment strategy warrant further investigation.

## 4 Conclusion

Our findings elucidate a previously unidentified mechanism of STING‐mediated IFN‐γ production in γδ T cells while highlighting the importance of the STING–IFN‐γ pathway in early tumor immunity. Furthermore, we elucidated the inhibitory role of TGF‐β in γδ T cell function during late‐stage tumor development. These findings provide valuable insights into the complex interplay among STING, TGF‐β, and γδ T cells in the tumor microenvironment and pave the way for developing innovative immunotherapeutic strategies for cancer. Our findings also highlight the significance of combining TGF‐β blockade and STING activation as targets for cancer treatment.

## 5 Experimental Section

### Research Ethics

Mouse experiment protocols were approved by the Laboratory Animal Ethics Committee of Jinan University (IACUC‐20210115‐14). Human data collection was approved by the Guangdong Provincial People's Hospital (No. GDREC2019162H(R1)), Guangzhou, China. Patients provided informed consent for data collection.

### Mice

The mice used in this study were 6–8 weeks old and had a C57BL/6J background. *Tmem173* ^−/−^ (*Sting1* ^−/−^) mice^[^ ^30^ ^]^ were obtained from Dr. Zhengfan Jiang (Peking University, Beijing, China), whereas *cGAS^−/−^* mice were obtained from Dr. Fuping You (Peking University, Beijing, China). SCID mice were purchased from Charles River Company in Zhejiang, China. B6.Cg‐Tg (CD2‐icre)4Kio/J (hCD2‐iCre) and *TCRδ^−/−^* mice were purchased from The Jackson Laboratory (Bar Harbor, ME). Eomes‐flox, and T‐bet‐flox mice^[^ ^37^ ^]^ were provided by Zhongjun Dong from Tsinghua University (Beijing, China). *Sting1^S365A/S365A^* mutant mice^[^ ^36^ ^]^ were obtained from Dr. Nan Yan (UT Southwestern Medical Center, USA). Sex‐ and age‐matched animals were assigned randomly to different groups. All mice were housed under specific pathogen‐free conditions.

### Cell Lines

The *cGAS^−/−^* B16 cell line was obtained from Dr. Yangxin Fu (Tsinghua University). B16 cells were obtained from the National Collection of Authenticated Cell Cultures (Shanghai, China). Cells were cultured in Dulbecco's modified Eagle's medium (DMEM) supplemented with 10% (vol/vol) fetal bovine serum (FBS), 10 mM HEPES, 2 mM L^−1^ L‐glutamine, 1 mM sodium pyruvate, 100 U mL^−1^ penicillin and 100 mg mL^−1^ streptomycin at 37 °C with 5% CO_2_. Mycoplasma tests were conducted monthly and confirmed to be negative.

### scRNA‐seq

The γδ T cells were isolated from tumor tissues of B16F0 tumor‐bearing mice on day 9 (*n* = 25) and the lymph nodes of naïve mice (*n* = 20). Immediately after sorting, the γδ T cells were run on a 10× Chromium platform (10× Genomics) and then subjected to library preparation by Shanghai Ouyi Biomedical Technology Co., Ltd., following the protocol recommended for the Chromium Single Cell 3′ Reagent Kit (v.3.1). The Fastq files for scRNA‐seq data from the Chromium 10× libraries were aggregated using the Cell Ranger (version 3.1.0) software (10× Genomics) to read single‐cell barcodes and perform genome mapping, in addition to unique molecular identifier (UMI) transcript quantification. All scRNA sequencing reads were referenced to the mouse genome mm10. Gene expression matrices were further analyzed with the R package Seurat (v4.3.0) via RStudio (v4.2.3).^[^ ^38^ ^]^

### Reagents

The InVivoMAb anti‐mouse TCR γ/δ (Clone: UC7‐13D5), purified anti‐mouse TCR Vγ4 mAb (Clone: UC3‐10A6), InVivoMAb anti‐mouse CD3ε (Clone:145‐2C11), and InVivoMAb anti‐mouse CD28 (Clone: PV1) were purchased from BioXcell (NH, USA). The PerCP/Cy5.5 anti‐mouse IL‐17A antibody (Clone: 17F3), FITC anti‐mouse CD8a (Clone: 53.6.7), PE‐anti‐mouse TCR γ/δ antibody (Clone: UC7‐13D5), PE anti‐mouse TCR Vγ2 antibody (Clone: UC3‐10A6), PE/Cy7 anti‐mouse IFN‐γ antibody (Clone: XMG1.2), PE‐Cy7‐ conjugated anti‐mouse CD3 mAb (Clone: 145‐2C11), PE/CY7 anti‐human CD4 antibody (Clone: RPA‐T4), and APC‐Cy7 anti‐mouse CD3 antibody (Clone: 17A2) were purchased from Biolegend (San Diego, CA, USA). The FITC anti‐mouse CD4 antibody (Clone: GK1.5) was purchased from Sungen (Tianjin, China). The BV421 mouse IgG1, k Isotype (Clone: X40), purified mouse anti‐human TCR γδ, PE anti‐human TCR γ/δ antibody, PE/CY7 anti‐human IFN‐γ antibody, and APC‐H7 anti‐human CD3 antibody (Clone: SK7) were purchased from BD Biosciences (MA, USA). The anti‐mouse IFN‐γ (Clone:XMG1.2), anti‐mouse Eomes, (Clone: Dan11mag), BV421 anti‐human IFN‐γ antibody (Clone: 4S.B3), and BV421 anti‐mouse CD45 antibody (Clone: 30‐F11) were purchased from Invitrogen (MA, USA). The cell proliferation dye eF450 was purchased from eBioscience. Anti‐mouse β‐actin mouse mAb (Clone: 6G3), anti‐mouse cGAS (D3O8O) rabbit mAb, anti‐human cGAS (D1D3G) rabbit mAb, TBK1/NAK (E8I3G) rabbit mAb, STING (D2P2F) rabbit mAb, p‐IRF3(S396) (4D4G) antibody, and p‐TBK1/NAK(S172) (D52C2) were purchased from Cell Signaling Technology

(MA, USA). The remaining reagents included PerCP/Cyanine5.5‐Lag3 (Clone: C9B7 W Catalog #125 211; Biolegend), eFluor 450‐Tim3 (Clone: 8B.2C12, Catalog # 48‐5871‐82; Invitrogen), decitabine (Cat#S1200, CAS#:2353‐33‐5; Selleckchem), TGF‐β1 (304‐B3‐001; R&D Systems), and TGF‐βRII inhibitor (HY‐12043; MCE). Antibody details are presented in the Supporting Information.

### Tumor Models

B16‐F0 melanoma cells (4 × 10^5^) were mixed with Vγ4 γδ T cells (1 × 10^5^) and injected subcutaneously (s.c.) into mice. Tumor growth was measured with a digital caliper and recorded daily as described previously.^[^ ^2^, ^31^ ^]^ For the B16 melanoma lung metastasis experiments, mice were injected intravenously with 2 × 10^6^ cells, and on day 1 after tumor inoculation, 4 × 10^6^ activated γδ T cells were transferred into mice via retroorbital intravenous injection. Lungs were harvested on day 28. Administration of compounds, measurements of tumors, and counting of lung tumors were performed in a blinded fashion.

### Xenograft Mouse Tumor Models

Male C57BL/6 WT mice were injected s.c. with 4 × 10^5^ B16 cells. Tumor growth was measured every 2–3 days after tumor inoculation using digital calipers. Mice were sacrificed on days 9, 13, 17, and 21 after inoculation, and tumors were excised and weighed. For γδ T cell adoptive transfer experiments, at 15 days post‐inoculation of B16 cells (4 × 10^5^ cells/mouse) into *TCRδ^−/−^* mice, γδ T cells were pretreated with cGAMP (5 µg mL^−1^), TGFβR inhibitor (2 µM), and cGAMP with TGFβR inhibitor for 24 h and were adoptively transferred i.v. into the tumor‐bearing TCRδ^−/−^ mice. Tumor growth and weight were determined.

### Confocal Immunofluorescence Microscopy

For histopathology and immunofluorescence staining, human cancer tissue samples were obtained from patients at Guangdong Provincial People's Hospital. The lung tumor tissues were fixed in 4% paraformaldehyde (Electron Microscopy Sciences), followed by dehydration using an ethanol and xylene concentration gradient, embedding in paraffin, and sectioning at a thickness of 5 µm using a paraffin slicing machine (HistoCore AUTOCUT; Leica). After deparaffinizing and antigen retrieval, the tissue slices were incubated with the primary antibody (mouse anti‐human TGF‐β1 (1:200, R&D, MAB2402) and rabbit anti‐human STING (1:200, CST, 13647S) in blocking solution (10% bovine serum albumin [BSA], 0.1% Triton‐X‐100 in PBS) overnight at 4 °C. The next day, after a thorough wash with PBS plus 0.1% Triton‐X‐100 for 5 min 3 times, the slices were incubated with the secondary antibody (Alexa 546‐conjugated donkey anti‐rabbit (1:1000) and Alexa 488‐conjugated donkey anti‐mouse antibody (1:1000) (ThermoFisher Scientific) and APC‐conjugated anti‐human TCRγδ antibody (Biolegend, clone B1) for 2 h at room temperature. Slices were mounted with an anti‐fade mountant containing DAPI (ThermoFisher Scientific) and imaged under a confocal microscope (Olympus FV3000, Japan).

### In Vivo Evaluation of the Antitumor Activity of Vγ9Vδ2 T Cells in SCID Mice

GFP‐A549 cells (5  ×  10^6^ cells/mouse) were injected s.c. into SCID mice. Subsequently, the lung cancer‐bearing mice were divided randomly into 3 groups (*n*  = 10) on day 22. PBS (100 µL), allogeneic Vγ9Vδ2 T cells, or diABZi‐C3‐treated allogeneic Vγ9Vδ2 T cells (5 × 10^6^ cells/mouse; cells with > 90% purity were resuspended in 100 µL PBS) were administered i.v. on days 22, 27, 32, 37, 42, and 47. For such mouse experiments, infused Vγ9Vδ2 T cells were prepared from the same donor by using frozen huPBMCs. Mice with subcutaneous tumors > 17 mm in diameter were euthanized and counted as having died. Tumor weight was measured on day 58.

### In Vitro DNMT1 Inhibitor Treatment

Mouse γδ T cells were sorted and expanded as described previously4^2^. For functional assays, Day 6‐Vγ4 γδ T cells were used, and purity was confirmed (>90%). Vγ4 γδ T cells were treated for 24 h with 50 nM decitabine (Selleckchem, CAS#:2353‐33‐5), 0.5 ng mL^−1^ TGF‐β1 (R&D Systems, 304‐B3‐001), or vehicle control (DMSO) at indicated concentrations.

### Cell Preparation, Activation, Treatment, and Staining

Mouse γδ T cells were sorted and expanded as described previously.^[^ ^39^ ^]^ For functional assays, Day 6‐γδ T cells were treated with the STING agonist DMXAA (10 µg mL^−1^) for 6 h with or without a TBK1 inhibitor (15 µM) for 6 h or an NF‐κB inhibitor (20 µM) for 6 h. To expand human Vδ2 γδ T cells, PBMCs were isolated from healthy donors or untreated patients with lung cancer using a Ficoll‐Paque‐based density gradient centrifugation protocol. The cells were cultured in Roswell Park Memorial Institute (RPMI)‐1640 medium supplemented with 10% FBS and antibiotics. To activate cells, ZOL (50 µM working concentration, Sigma) and recombinant IL‐2 (100 IU mL^−1^) were added to the culture medium on day 0. After 12–14 days of culture, the cells were treated with a human STING agonist (diABZI compound C3) at a final concentration of 100 nM for 6 h. For intracellular cytokine staining, protocols described in the previous publications were used.^[^ ^31^, ^37^ ^]^ FACS was performed with BD FACSVerse, and data were analyzed using FlowJo v.10.

### In Vitro Killing Assay

B16 cells, LLC cells, and MC38 cells (8000 cells/well) were labeled with carboxyfluorescein succinimidyl ester (CFSE; 5 µM). Mouse splenocytes were labeled with CFSE (0.25 µM) as the internal control. Subsequently, B16, LLC, or MC38 cells and splenocytes were mixed with expanded γδ T cells (E: T = 20) for different time points. The proportion of specific killing was calculated as follows: 100 × (% of CFSE^lo^ – % of CFSE^hi^)/% of CFSE^lo^.

### Real‐Time PCR for Gene Transcription

Total RNA was extracted from cells and reverse‐transcribed with a Takara reverse transcription kit. The primer sequences are presented in the Supporting Information. Real‐time PCR was performed with a Bio‐Rad CFX Connect cycler.

### Immunoblotting Analysis

Cells were collected and lysed using RIPA Lysis Buffer (Beyotime), complemented with a complete EDTA‐free protease inhibitor cocktail (Roche) and phosphatase inhibitor cocktail (Bimake). Lysates were incubated for 30 min on ice followed by centrifugation at 4 °C for 30 min. Supernatants were collected, and protein contents were quantified using a bicinchoninic acid (BCA) protein assay kit (Thermo Pierce). Cell total proteins (20–40 µg) were separated on 12% SDS‐PAGE gels and transferred to a 0.22‐µm polyvinylidene difluoride (PVDF) membrane with the PowerPac wet‐blot system (Bio‐Rad). Membranes were incubated in blocking solution (5% bovine serum albumin) for 1 h and probed with primary antibodies against the following proteins: anti‐mouse cGAS (D3O8O) rabbit mAb (CST, #31 659), anti‐human cGAS (D1D3G) rabbit mAb (CST, #15 102), TBK1/NAK (E8I3G) rabbit mAb (CST, #38 066 H/M), anti‐TBR2/Eomes antibody (EPR21950‐241) (Abcam, ab216870 H/M), anti‐IRF3 antibody (EPR2418Y) (Abcam, ab68481 H/M), STING (D2P2F) rabbit mAb (CST, #13 647 H/M), anti‐mouse beta‐actin mouse mAb (Proteintech, 2D4H5), or anti‐human GAPDH mouse mAb (Proteintech, 1E6D9) at 4 °C overnight. After 6 washes in TBST for 1 h, the membranes were stained with secondary antibodies conjugated to horseradish peroxidase at a dilution of 1:3000 (CST, goat anti‐rabbit, or goat anti‐mouse) at room temperature for 2 h. Enhanced chemiluminescence (Millipore) was used to assess the chemiluminescence signals on the Bio‐Rad ChemiDoc MP Gel imaging system (Bio‐Rad). Images were quantified using ImageJ analysis software.

### Enzyme‐Linked Immunosorbent Assay (ELISA)

The mouse IFN‐β ELISA kit was purchased from Cusabio (Wuhan, China). The mouse IFN‐α and IFN‐γ ELISA kit was purchased from Biolegend (San Diego, CA, USA). Experiments were performed following the manufacturer's instructions.

### Luciferase Reporter Assay

HEK293T cells seeded on a 24‐well plate were transiently transfected with 50 ng of the luciferase reporter plasmid and equal amounts of various expression plasmids or empty control plasmids. As an internal control, 10 ng pRL‐TK was transfected. After 24 h, reporter gene activity was analyzed using the Dual‐Luciferase Reporter 1000 Assay System (Promega, E1960) and measured with a TD‐20/20 Luminometer (Turner Designs) according to the manufacturer's instructions.

### Mass Spectrometry

To identify proteins that may interact with Eomes, samples pulled down by the Eomes antibody were subjected to mass spectrometry analysis. To identify Eomes phosphorylation sites, Eomes were precipitated from γδ T cells, and the lysate (1.5% SDS/100 mM Tris‐Cl) was added to the cell sample and mixed thoroughly. The cell homogenate was centrifuged to obtain the supernatant precipitated by acetone precipitation. A redissolve solution (8 m urea/100 mM Tris‐Cl) was added to the protein precipitate. For in‐solution digestion, 100 µL of each sample was reduced by adding 100 mM DTT until a final concentration of 20 mM was achieved. The sample was then incubated at 37 °C for 60 min and alkylated by incubation with 50 mM IAA in the dark at room temperature for 30 min. Finally, overnight digestion with trypsin was performed at a 1:100 trypsin: total protein ratio at 37 °C. The reaction was terminated by placing the sample in a −20 °C freezer. An EASY‐nLC 1200 system was coupled with the Orbitrap Exploris 480 LC‐MS/mass spectrometer. Peptide fractions were injected under trapping conditions (trap column: 3 µm, 120 Å, 100 µm × 20 mm) using an EASY‐nLC 1200 system equipped with a separation column (2 µm, 120 Å, 750 µm × 150 mm). The gradient ran at 300 nL min^−1^ from 5% B (0.1% formic acid in acetonitrile) to 80% B over 50 min, increased to 95% B to wash the column, and re‐equilibrated at 5% B from 50 min to 65 min. Each cycle comprised an Orbitrap‐MS spectrum acquisition for 20 ms (mass range 350–1800 Da), followed by the acquisition of up to 20 MS/MS spectra (mass range 100–2000 Da) of MS peaks at the above intensity per full cycle. The higher‐energy collisional dissociation was set to 30. The dynamic exclusion time for repeated ion collection was set to 35 s. Data were analyzed using the Protein Discovery software (Thermo). The database used for the search was the mouse proteome reference database in UniProt. The main retrieval parameters are as follows: variable modification oxidation (M), acetyl (protein N‐term), fixed modification carbamidomethyl (C), phosphorylation (STY). The retrieval results were screened based on the protein and peptide levels of 1% FDR.

### ChIP and ChIP‐Seq

ChIP of crosslinked chromatin was performed as described previously, with minor modifications.^[^ ^37^ ^]^ Cell extracts from 50 × 10^7^ cells were sonicated 12 times using the S‐4000 Sonifier (Misonix, Farmingdale, NY) with 30‐s pulses, to obtain DNA fragments between 200 and 500 bp in length. Chromatin extracts were prewashed with 50 µL of magnetic beads (Invitrogen) for 1 h at 4 °C in a rotating wheel. Precipitation was then carried out overnight at 4 °C using 10 µg of anti‐Eomes (Abcam ab216870) or anti‐T‐bet (Abcam ab275959) antibodies complexed with 50 µL of magnetic beads. ChIP‐seq was performed at Wuhan Seqhealth Technology Co., Ltd. (Wuhan, China). For ChIP, PCR analyses were performed using the following primers:F1: 5′‐TTGAACTTGATGGGGGAAAC‐3′; R1: 5′‐CACAGCAGAAATCACTTCAGGA‐3′.

### Statistical Analysis

For FACS, real‐time PCR, and the killing assay, statistical significance was determined using a two‐tailed unpaired Student's *t*‐test. For tumor size comparison, a two‐way analysis of variance (ANOVA) was applied. All statistical analyses were performed using GraphPad Prism 6.0 for Windows (GraphPad Software Inc.). *, *p* ≤ 0.05, **, *p *≤ 0.01, ***, *p* ≤ 0.001.

## Conflict of Interest

The authors declare no conflicts of interest.

## Author Contributions

J.L., S.W., and Q.Y. contributed equally to this work. J.L., S.W., and Q.Y. performed most of the experiments. Q.F. and T.L. assisted in processing mouse samples for flow cytometry analysis. Q.Z., R.G., and Y.Z. performed scRNA‐seq analysis. S.Y. and G.Y. helped manage the mouse cohorts. X.B., S.L., and Y.Z. helped with human samples. Z.J., H.Z., H.X., D.K., G.S., Z.G., and L.L. helped revise the manuscript and performed some animal analyses. L.L., F.Y., J.H., and Z.Y. wrote the manuscript and supervised the study.

## Supporting information



Supporting Information

## Data Availability

All data supporting the findings of this study are available within the article and its supporting information files and from the corresponding author upon reasonable request. The raw data for mouse and human γδ T cell RNA‐seq have been deposited at SRA database under accession number PRJNA877422. The raw data of mouse γδ T cell ChIP‐seq have been deposited at SRA database under accession number PRJNA877450. The data that support the findings of this study are available from the corresponding author upon reasonable request.
