# Supplementary material for: γδ T Cell‐mediated Tumor Immunity is Tightly Regulated by STING and TGF‐β Signaling Pathways
Source: Adv Sci (Weinh). 2024 Nov 21;12(2):2404432. doi: 10.1002/advs.202404432 (PMC11727375; doi:10.1002/advs.202404432)
Supplement: Supplementary file 1 — Supporting Information [file ADVS-12-2404432-s001.docx]

Supplemental Figures:

**Figure S1. Endogenous STING promotes γδ T cell mediated tumor inhibition.**

(A-D) LLC, MC38 and B16F0 cells (target cells) were labeled with high concentration of CFSE (5 μM) and splenocytes were labeled with low concentration of CFSE (0.25 μM) as internal cell number control. WT or *Sting1^-/-^* γδ T cells were expanded for 6 days and treated with DMXAA for 6 h as effector cells. Different numbers of effector γδ T cells were mixed separately with the target cells. Cells were co-cultured for 24h and remaining target cells were analyzed by FACS. Representative histograms were shown (A) and statistics of specific killing of triplicates was shown (B-D). Data are mean ± SEM. **P* ≤ 0.05, ***P* ≤ 0.01, ****P* ≤ 0.001; ns, not significant, two-tailed unpaired t test. Data are representative of three independent experiments.

**Figure S2. STING activation induced a comprehensive Th1-like response in mouse γδ T cells while IFN-γ was significantly inhibited in mouse αβ T cells.**

(A) WT Vγ4 γδ T cells were expanded for 6 days (purity >90%) and were treated with DMXAA for 6h. Cells were collected for RNA-seq, clustered heatmap for interferon-related genes with altered expression was shown. (B) Purified mouse CD4^+^ T cells were expanded for 6 days and were treated with DMXAA for 6h. Cells were collected for RNA-seq, clustered heatmap for interferon-related genes with altered expression was shown. (C) Purified mouse CD8^+^ T cells were expanded for 6 days and were treated with DMXAA for 6h. Cells were collected for RNA-seq, clustered heatmap for interferon-related genes with altered expression was shown.

**Figure S3. TNF-α rather than IFN-γ was upregulated upon DMXAA stimulation in CD8 T cells.**

(A-C) WT CD8 T cells were expanded for 6 days and treated with DMSO, DMXAA for 6h and cGAMP for 24 h, cells were collected for intracellular cytokine staining and FACS analysis. Gating strategy (A), representative staining and statistical plot of IFN-γ (B) and TNF-α (C) expression in CD8 T cells were shown. Data are mean ± SEM. ****P* ≤ 0.001; ns, not significant, two-tailed unpaired t test. Data are representative of three independent experiments.

**Figure S4. STING activation does not change NKG2D levels in Vγ4 γδ T cells.**

(A-B) WT Vγ4 γδ T cells were expanded for 6 days and treated with DMSO, DMXAA or cGAMP for 6 h, cells were collected for NKG2D staining. (A) Gating strategy. (B) Representative staining and statistical plot were shown. Data are representative of three independent experiments.

**Revised Figure S5. γδ T-mediated rejection of B16 tumors requires cGAMP from tumor cells.**

(**A-B**) Vγ4 γδ T cells (1 × 10^5^ cells) from WT or *cGAS^-/-^* mice were expanded for 5–6 days, mixed with B16F0 cells (4 × 10^5^ cells), and co-injected into the flank of *TCRδ^-/-^* mice; tumor growth was monitored (n = 9 per group) (**A**). WT mice or *TCRδ^-/-^* mice were inoculated with B16 and *cGAS^-/-^* B16 cells and monitored for tumor growth (n = 9 per group) (**B**). Data are mean ± SEM. ***P ≤ 0.001; ns, not significant, two-tailed unpaired *t-*test. Data are representative of three independent experiments.

**Revised Figure S6. DMXAA and cGAMP promotes γδ T cell IFN-γ production.**

(A-B­) WT or *Sting1^-/-^* Vγ4 γδ T cells were expanded for 6 days and treated with DMSO, DMXAA for 6 h or cGAMP for 24 h, cells were collected for intracellular cytokine staining and FACS analysis. Representative staining (A) and quantification (B) of IFN-γ in Vγ4 γδ T cells was shown. Data are mean ± SEM. ***P* ≤ 0.01; ns, not significant, two-tailed unpaired t test. Data are representative of three independent experiments.

**Revised Figure S7. S365 site of STING is important for IFN-γ production in γδ T cells.**

(A-B­) WT or *Sting^S365A^* Vγ4 γδ T cells were expanded for 6 days and treated with DMSO, DMXAA for 6 h or cGAMP for 24 h, cells were collected for intracellular cytokine staining and FACS analysis. Representative staining (A) and quantification (B) of IFN-γ in Vγ4 γδ T cells was shown. Data are mean ± SEM. ***P* ≤ 0.01; ns, not significant, two-tailed unpaired t test. Data are representative of three independent experiments.

**Revised Figure S8.** **cGAS-STING signaling induced γδ T cell IFN-γ production via TBK1.**

(A) WT or cGAS^-/-^ γδ T cells were expanded for 6 days and treated with DMXAA for 6 h as effector cells. DMXAA-treated cells were re-stimulated with PMA and ionomycin in the presence of GolgiPlug for 4 h and intracellular IFN-γ were stained. Quantification of IFN-γ in Vγ4 γδ T cells was calculated. (B-D) WT Vγ4 γδ T cells were expanded for 6 days (purity >90%) and were treated with DMXAA with or without Amlexanox (15 μM) for 6h. RNA was extracted for Real-time PCR for *Ifna* (A), *Ifnb* (C) and *Ifng* (D). Data are mean ± SEM. **P* < 0.01, ***P* < 0.01 ****P* < 0.001 (two-tailed unpaired t test). Data are representative of three independent experiments.

**Revised Figure S9.** **T-bet is dispensable for DMXAA and cGAMP-induced IFN-γ in Vγ4 γδ T cells.**

(A-B) Vγ4 γδ T cells from *T-bet^fl/fl^* or *CD2-cre-T-bet^fl/fl^* mice were expanded for 6 days (purity >90%) and were treated with DMXAA or cGAMP for 6h, cells were re-stimulated with PMA and ionomycin in the presence of GolgiPlug for 4 hours and intracellular IFN-γ were stained and analyzed. Representative staining (A) and quantification (B) of IFN-γ in Vγ4 γδ T cells were shown. Data are mean ± SEM. **P* ≤ 0.05, ***P* ≤ 0.01, two-tailed unpaired t test. Data are representative of three independent experiments.

**Supplementary materials**

Primers for Real-time PCR

| Gene | Forward primer (5’-3’) | Reverse primer (5’-3') |
| --- | --- | --- |
| *Ifna* | TACTCAGCAGACCTTGAACCT | CAGTCTTGGCAGCAAGTTGAC |
| *Ifnb* | CAGCTCCAAGAAAGGACGAAC | GGCAGTGTAACTCTTCTGCAT |
| *Ifng* | AGACAATCAGGCCATCAGCA | CAACAGCTGGTGGACCACTC |
| *Actb* | AACAGTCCGCCTAGAAGCAC | CGTTGACAT CCGTAAAGACC |

Information for antibodies

| Name&Clone | Company | Cat. | LOT. |
| --- | --- | --- | --- |
| InVivoMAb anti-mouse TCR γ/δ（UC7-13D5） | BioXcell | BE0070 | 762421J1 |
| purified anti-mouse TCR Vg4 mAb, (Clone UC3-10A6) | BioXcell | 132 |  |
| InVivoMab anti-mouse CD3ε(Clone 145-2C11) | BioXcell | BE0001-1 | 759120J1 |
| InVivoMab anti-mouse CD28（Clone:PV1） | BioXcell | BE0015-1 | 639918N1 |
| PERCP/Cyanine5.5 anti-mouse IL17 anti-body (17F3), | Biolegend | 506920 | B319621 |
| FITC anti-mouse CD8a  (53.6.7) | Biolegend | 100706 | B277418 |
| [FITC anti-mouse CD4 Antibody](https://www.biolegend.com/en-us/products/fitc-anti-mouse-cd4-antibody-248)  (GK1.5) | Sungen Biotech | M100430-02E | AAZ06 |
| PE-anti-mouse TCR γ/δAntibody (UC7-13D5) | Biolegend | 107508 | B285402 |
| PE anti-mouse TCR Vγ2 Antibody (UC3-10A6), | Biolegend | 137706 | B289538 |
| PE/Cy7 anti-mouse IFN-γ Antibody (XMG1.2), | Biolegend | 505826 | B311730 |
| PE-Cy7- conjugated anti-mouse CD3 mAb (145-2C11) | Biolegend | 100320 | B316240 |
| Vadimezan-DMXAA | MCE | 117570-53-3 | #66992 |
| Amlexanox (TBK1 inhibitor) | invivogen | #inh-amx | #ALX-38-01 |
| STING agonist diABZI Compound3 | Probechem | 2138299-3438 | PC-35785 |
| BV421 Mouse Anti-Human STING(clone：T3-680) | BD Biosciences | 564966 | 0247082 |
| BV421 Mouse IgG1,k Isotype (clone：X40) | BD Biosciences | 562438 | 0136398 |
| Purified Mouse Anti-Human TCR γδ | BD Biosciences | 555715 |  |
| Anti-Mouse IFN gamma，eBioscience eF450(Clone：XMG1.2) | Invitrogen | 48-7311-82 | 2026274 |
| PE anti-human TCR γ/δ Antibody | BD Biosciences | 555717 | 5267944 |
| PECY7 anti-human IFN γAntibody | BD Biosciences | 557643 | 7202642 |
| Anti-Mo EOMES gamma，eBioscience eF450(Clone：Dan11mag) | Invitrogen | 48-4875-82 | 21282290 |
| anti-mouse β-actin mouse mAb(Clone：6G3) | Cell Signaling Technology | KM9001 | I0112 |
| anti-human GADPH mouse mAb | proteintech | 1E6D9 |  |
| APC-conjugated anti-mouse T-bet（Clone:4B10） | Biolegend | 644814 | B279042 |
| APC-H7 anti-human CD3 antibody(Clone:SK7) | BD Biosciences | 560176 | 1021392 |
| BV421 anti-human IFN-γ antibody（Clone:4S.B3） | Invitrogen | 2023-10-29 | 2022936 |
| PECY7 anti-human CD4 antibody（Clone:RPA-T4） | Biolegend | 300512 | B269744 |
| anti-mouse cGAS (D3O8O) Rabbit mAb | Cell Signaling Technology | 31659S | 3 |
| anti-human cGAS (D1D3G) Rabbit mAb | Cell Signaling Technology | 151023 | 4 |
| TBK1/NAK (E8I3G) Rabbit mAb | Cell Signaling Technology | 38060S | 1 |
| STING (D2P2F) Rabbit mAb | Cell Signaling Technology | 13647S | 5 |
| anti-IRF3 antibody （EPR2418Y） | Biotechnology | #G2310 |  |
| BV421 anti-mouseCD45 antibody（Clone:30-F11） | Invitrogen | 48-0451-82 | 197161 |
| APC-cy7 anti-mouse CD3 antibody（Clone:17A2） | Biolegend | 100222 | B324939 |
| P- IRF3(S396)(4D4G)antibody | Cell Signaling Technology | #4947S | 7 |
| Anti-Rabbit IgG,Light Chain | Proteintech | 20000234 | SA00001-7-L |
| Affinipure Goat Anti-Mouse | Proteintech | 20000002 | SA00001-1 |
| P-TBK1/NAK(S172)(D52C2) | Cell Signaling Technology | 54837 | 11 |
| 2'3'-cGAMP | Invitrogen | 1441190-66-4 | tlrl-nacga23-02 |
| Carbenoxolone disodium | Tocris | 7421-40-1 | 3096 |
| DCPIB | Tocris | 827-70-0 | 1540 |
| ELISA MAX™ Deluxe Set Mouse IFN-γ | Biolegend | 430804 |  |
| Mouse Interferon β,IFN-β/IFNB ELISA Kit | Cusabio | CSB-E04945m |  |
| ELISA MAX™ Deluxe Set Mouse IFN-α1 | Biolegend | 447904 |  |
